# Systematic review and meta-analysis of vaginal natural orifice transluminal endoscopic surgery hysterectomy versus vaginal hysterectomy for benign indications

**DOI:** 10.1016/j.xagr.2024.100355

**Published:** 2024-05-10

**Authors:** Greg J. Marchand, Hollie Ulibarri, Amanda Arroyo, Madison Blanco, Daniela Gonzalez Herrera, Brooke Hamilton, Kate Ruffley, Ali Azadi

**Affiliations:** 1 Marchand Institute for Minimally Invasive Surgery, Mesa, AZ (Marchand, Ulibarri, Arroyo, Blanco, Herrera, Hamilton, and Ruffley); 2 University of Arizona, College of Medicine, Phoenix, AZ (Azadi); 3 Creighton University, School of Medicine, Phoenix, AZ (Azadi)

**Keywords:** TVH, vaginal hysterectomy, vaginal surgery, vNOTES

## Abstract

**Objective:**

As the second most common surgery performed on women in the United States, hysterectomy techniques are constantly examined for validity and superiority. The vaginal natural orifice transluminal endoscopic surgery (vNOTES) has increased in popularity since the first vNOTES hysterectomy was performed in 2012. We sought out to evaluate the safety and effectiveness of hysterectomy by vNOTES compared to conventional vaginal hysterectomy for various benign indications.

**Data sources:**

We searched Scopus, Medline, PubMed, ClinicalTrials.Gov, and the Cochrane Library. Our search included all studies from each respective database's inception until September 1, 2023.

**Study eligibility criteria:**

We included eligible studies that compare vNOTES hysterectomy versus conventional vaginal hysterectomy for various benign indications, and included at least one of our preselected outcomes. The main outcomes were estimated blood loss (mL), operation time (min), length of hospital stay (d), Visual Analogue Scale pain score at Day 1, intraoperative complications, and postoperative complications.

**Study appraisal and synthesis methods:**

We analyzed data of our continuous outcomes using RevMan 5.4.1. Continuous outcomes were analyzed using mean difference (MD) and 95% confidence intervals (CIs) under the inverse variance analysis method. We assessed the quality of the studies using the ROBINS-I assessment tool.

**Results:**

We found 4 eligible studies to include in our analysis. Surgeon declared estimated blood loss was found to be similar in both groups (MD=−44.70 [−99.97, 10.57]; *P*=.11). Also, the total length of hospital stay (in days) was found to be comparable in both groups (MD=−0.16 [−1.62, 1.30]; *P*=.83). We also found no other statistically significant difference between hysterectomy by vNOTES and vaginal hysterectomy in other studied outcomes, including the duration of the operation, the Visual Analogue Scale Pain score after 1 day, intraoperative complications, and postoperative complications.

**Conclusion:**

vNOTES seems to be associated with a nonsignificant lower surgeon declared estimated blood loss. We found no other significant differences in hospital stay, intraoperative, or postoperative outcomes. Further studies may clarify if other differences in safety or efficacy exist.

**AJOG Global Reports at a Glance**

**Why was this study conducted?**

– The best technique for minimally invasive hysterectomy for benign disease is widely debated and extremely controversial in gynecology today.
– Our researchers wanted to compare vNOTES to vaginal hysterectomy for safety and efficacy.

**Key findings**

– Using all available data we found that the surgeon declared estimated blood loss and length of hospital stay were similar in both groups.
– We found no other statistically significant difference in any other studied outcome, including the duration of the operation, the pain Visual Analogue Scale score after one day, intraoperative complications, and postoperative complications.

**What does this add to what is known?**

– This study adds to the evidence that vNOTES hysterectomy is a valid technique of minimally invasive hysterectomy, and that vNOTES may be associated with similar intraoperative less blood loss and hospital stay to vaginal hysterectomy.

## Introduction

As the second most common surgery performed on women in the United States (behind cesarean delivery), hysterectomy techniques are constantly examined for validity and superiority.^1^ The most common indication for hysterectomy is benign gynecological disorders, which accounts for more than 90% of hysterectomies performed worldwide.^2^ According to recently published Cochrane analyses on this topic,^2^ it is most useful to divide hysterectomy approaches into conventional laparotomy techniques, and minimally invasive surgical techniques (MIS). From there, MIS includes laparoscopy, laparoscopically assisted vaginal hysterectomy, robot-assisted laparoscopic hysterectomy, and natural vaginal orifice transluminal endoscopic surgical hysterectomy (vNOTES).^2^ Another previously published Cochrane review in 2023 investigated the efficacy and safety endpoints of the various surgical hysterectomy procedures for benign gynecological diseases. This review concluded that the vaginal approach to hysterectomy was associated with shorter hospital stay, fewer infection rates, lower costs, and an earlier return to regular activities; however, it may be limited by the availability of surgeons having expertise in this technique, as well as the limitations of complex manipulation techniques, and inherent poor visualization of the anatomy.^3^^,^^4^

The natural orifice transluminal endoscopic surgeries (NOTES) are a modern evolution in the past few years in MIS. vNOTES has increased in popularity since it was first used to perform cholecystectomy and hysterectomy in 2007 and 2012, respectively.^5^^,^^6^ In vNOTES, rather than using the abdominal wall to access the anatomy (as is used for conventional laparotomy and laparoscopic procedures), surgeons utilize the natural vaginal orifice to reach the abdominal cavity and perform surgeries. The result is improved recovery and cosmesis, leaving a scar-free abdomen.^7^^,^^8^ Hysterectomy by vNOTES could, potentially, overcome many of the limitations of conventional vaginal hysterectomy and preserve the benefits of laparoscopic visualization and easier manipulation.

## Objectives

To the best of our knowledge, there is no systematic review and meta-analysis to date that have compared vaginal hysterectomy, and hysterectomy by vNOTES. Therefore, we conducted this study to investigate the safety and effectiveness of hysterectomy by vNOTES compared to the conventional vaginal hysterectomy.

## Methods

We utilized PRISMA as a guideline in conducting this systematic review and meta-analysis.^9^

### Search and information databases

We used the following search strategy in our search: ("Natural Orifice Endoscopic Surgery" OR NOTES OR vNOTES OR ("natural" AND "orifice" AND "endoscopy*")) AND (vaginal hysterectomy). Scopus, Medline, PubMed, ClinicalTrials.Gov, and the Cochrane Library were the utilized online databases. Our search included all studies from each respective database's inception until September 1, 2023.

### Selection criteria and eligibility criteria

We selected the included studies following 2 steps. First, 2 authors GM and AA screened titles and abstracts to select relevant studies that were evaluated to reach the final included articles based on our inclusion criteria. A third author AT resolved any conflict between both authors .We included studies which compared hysterectomy by vNOTES versus vaginal hysterectomy for any benign indications. Single-arm studies, articles that did include any of our selected outcomes, and secondary research such as systematic reviews and meta-analyses we excluded.

### Data extraction

Data from the eligible articles data was extracted manually onto spreadsheets by 2 authors (GM and AT) independently. We extracted the general information of the studies in addition to the baseline data of included women, including age, BMI, parity, the number of prior surgeries, and the number of prior cesarean deliveries. We extracted data of main primary outcomes, including estimated blood loss (ml), operation time (min), length of hospital stay (d), VAS score at Day 1, intraoperative complications, and postoperative complications. Finally, we retrieved the required data for the risk of bias assessment. A third author (HU) reviewed the extracted data from the 2 authors.

### Risk of bias assessment

The 4 studies we found that met our inclusion and exclusion criteria were all found to be observational studies. Accordingly, we used ROBINS-I tool to assess the risk of bias of observational studies.^10^

### Statistical Analysis

We analyzed data of our continuous outcomes using RevMan 5.4.1. Continuous outcomes were analyzed using mean difference (MD) and 95% confidence intervals (CIs) under the inverse variance analysis method. Dichotomous outcomes were analyzed using the RevMan software as well as the Open Meta Analyst software.^11^ We used odds ratios (OR) and 95% CIs. The heterogeneity among the studies was assessed by the p-value of the Chi-square test and the I^2^ test. Outcomes were considered heterogeneous if *P*<.1 or I^2^>50%. We tried to solve the inconsistency among data using subgroup analysis.^12^

## Results

### Summary of the included studies

**Figure 1:**
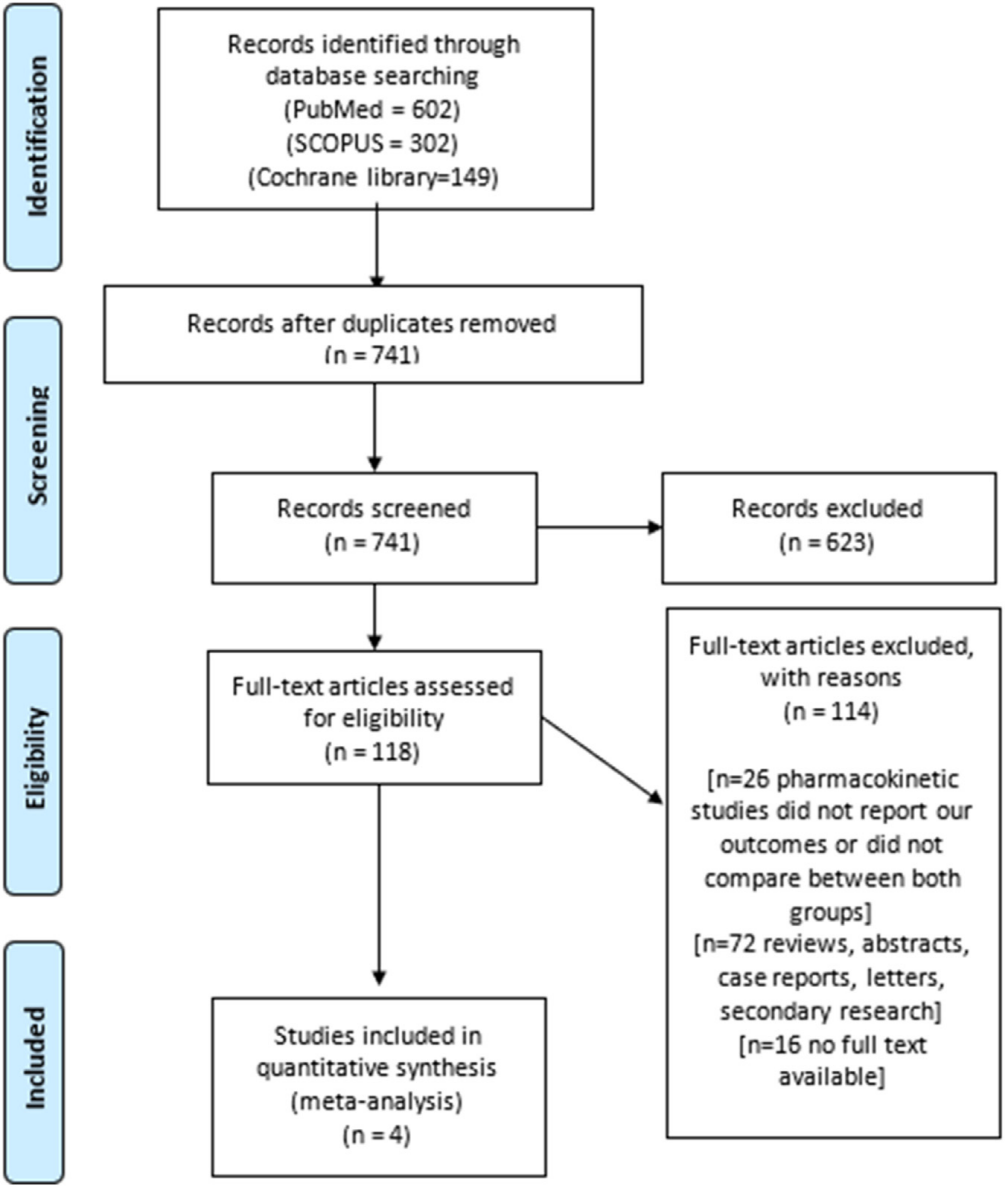
PRISMA flow diagram summarizing the results of our search.

The PRISMA flow diagram summarizes the results of our search (Figure 1). We ultimately included 4 eligible studies,^13^, ^14^, ^15^, ^16^ which in total included 373 patients. Of these, 160 women underwent hysterectomy by the vNOTES technique, and the other 213 women underwent hysterectomy by vaginal hysterectomy. The baseline characteristics of all of the included studies are illustrated in Table 1.

**Table 1:** The baseline characteristics of the included participants

Study	Study design	Sample size		Age, years (mean+SD)		BMI (kg/m^2^)		Parity (mean+SD)		Prior surgery, n (%)		Prior caesarean section	
		vNOTES	VH	vNOTES	VH	vNOTES	VH	vNOTES	VH	vNOTES	VH	vNOTES	VH
Aharoni 2021^13^	Retrospective cohort	65	70	59.93+12.0	59.5+7.7	29.40+5.9	29.26+3.6	3.40+1.7	3.8+1.8	14 (20%)	18 (27%)	NR	NR
Huang 2023^14^	Retrospective cohort	31	51	61.42±8.96	64.98±7.33	24.43±3.56	23.60±2.72	1.68±0.83	2.06±0.98	2 (6.5%)	4 (7.8%)	0 (0)	1 (2%)
Lee 2018^15^	Retrospective study	14	42	28.8+7.3	29.3+6.4	24.3+5.0	23.7+4.4	NR	NR	NR	NR	NR	NR
Merlier 2022^16^	Retrospective cohort	50	50	48.6±7.4	49.5±8.5	27.5±6.4	25.6±5.2	2.25+1.11	2.25+1.11	7 (14%)	12 (24%)	4 (8%)	8 (16%)

### Results of risk of bias assessment

We utilized the ROBINS-I to assess the risk of bias of observational studies. The overall risk of bias was moderate in all studies except Lee et al which was at serious risk of bias.^10^ Table 2 shows a detailed illustration of all domains.

**Table 2:** Risk of bias assessment of the included studies by ROBINS-I tool

Study	Bias due to confounding	Selection bias	Bias in classification of interventions	Bias due to deviations from intended intervention	Bias due to missing data	Bias in measurement of outcomes	Bias in selection of reported result
Aharoni 2021^13^	Moderate	Moderate	Low	Moderate	Low	Moderate	Low
Huang 2023^14^	Moderate	Moderate	Low	Moderate	Low	Moderate	Low
Lee 2018^15^	Serious	Moderate	Low	Low	Moderate	Moderate	Low
Merlier 2022^16^	Moderate	Moderate	Low	Low	Low	Moderate	Low

### Analysis of outcomes

#### Operative time (min)

**Figure 2:**
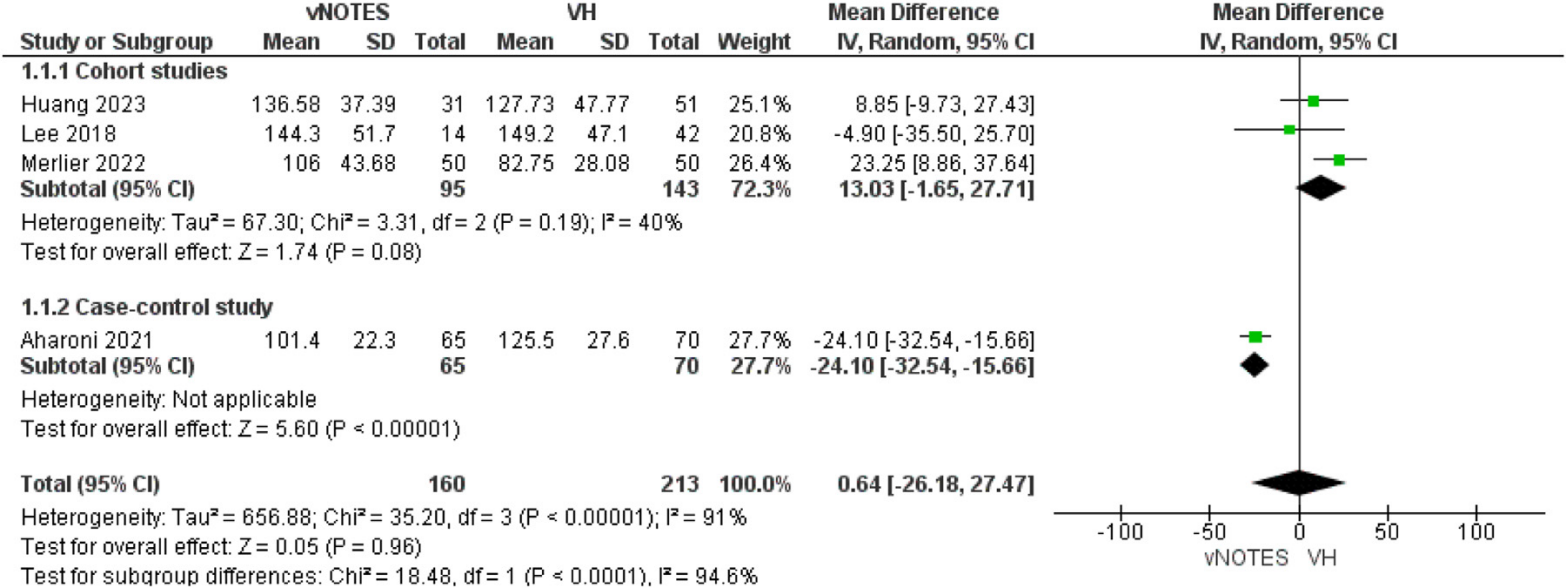
Meta-analysis of operative time in minutes.

All of the included studies reported this outcome.^13^, ^14^, ^15^, ^16^ We conducted subgroup analysis according to the study design of the included articles. Regarding the cohort subgroup, we found no significant difference in operative time between the 2 procedures (MD=13.03 [−1.65, 27.71]; *P*=.08). Data were homogeneous (*P*=.19); I²=40%. Concerning the case-control subgroup, Aharoni et al^13^ showed a decreased operation time in the vNOTES by MD=−24.10 [−32.54, −15.66]. We found that the overall operation time of conventional VH was comparable with that of vNOTES hysterectomy (MD=0.64 [−26.18, 27.47]; *P*=.96; Figure 2).

#### Surgeon declared estimated blood loss (mL)

**Figure 3:**
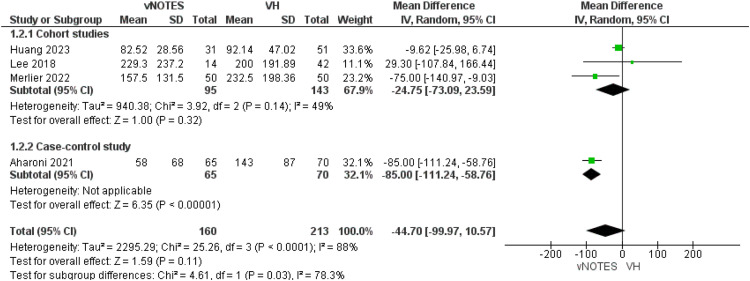
Meta-analysis of surgeon declared estimated blood loss, in mL.

All studies included the surgeon declared intraoperative blood loss.^13^, ^14^, ^15^, ^16^ The subgroup analysis of the cohort studies showed no significant difference between both procedures (MD=−24.75 [−73.09, 23.59]; *P*=.32). Data were homogeneous (*P*=.14); I²=49%. However, Aharoni et al in the case-control subgroup reported a decrease in the blood loss in patients allocated to the vNOTES group by MD=−85.00 [−111.24, −58.76]. Our overall analysis showed an overall similar blood loss in both procedures in both subgroups (MD=−44.70 [−99.97, 10.57]; *P*=.11; Figure 3).

#### Length of hospital stay (d)

**Figure 4:**
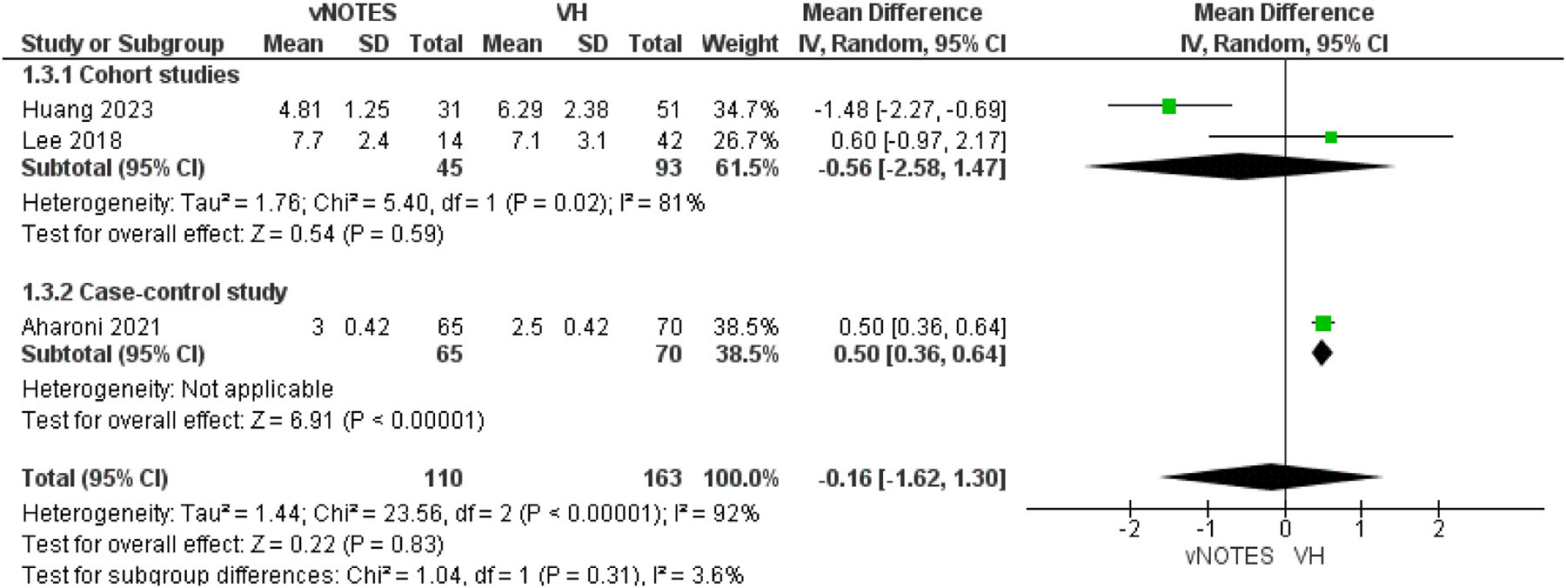
Meta-analysis of hospital stay, in days.

Three studies reported this outcome.^13^, ^14^, ^15^ The combined mean difference of the cohort subgroup showed similar hospitalization days in both groups (MD=−0.56 [−2.58, 1.47]; *P*=0.59). Aharoni et al in the case-control subgroup reported an increased hospital stay in the conventional hysterectomy group by MD=0.50 [0.36, 0.64]. The overall duration of hospitalization after both procedures was similar in both cohorts (MD=−0.16 [−1.62, 1.30]; *P*=.83; Figure 4). We could not solve the heterogeneity in this outcome and the random effect model was used.

#### VAS scores at day 1

**Figure 5:**
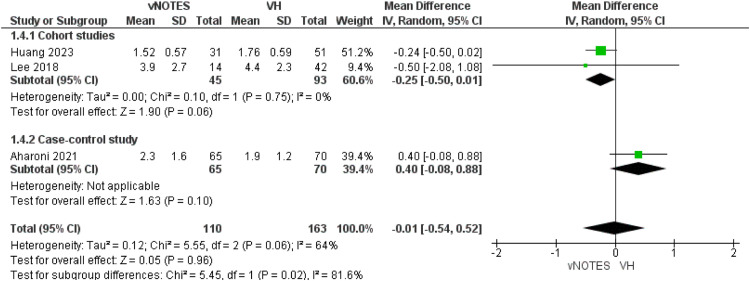
Meta-analysis of Visual Analogue Scale (VAS) pain scores on postoperative day 1.

This pain score was measured by 3 studies.^13^, ^14^, ^15^ The analysis of the cohort subgroup showed comparable VAS scores in both techniques (MD=−0.25 [−0.50, 0.01]; *P*=.06). Data were homogeneous (*P*=.75); I²=0%. The case-control subgroup also showed similar VAS scores MD=0.40 [−0.08, 0.88]. The overall measured VAS score at day 1 after both procedures was comparable (MD=−0.01 [−0.54, 0.52]; *P*=.96; Figure 5).

#### Intraoperative complications

**Figure 6:**
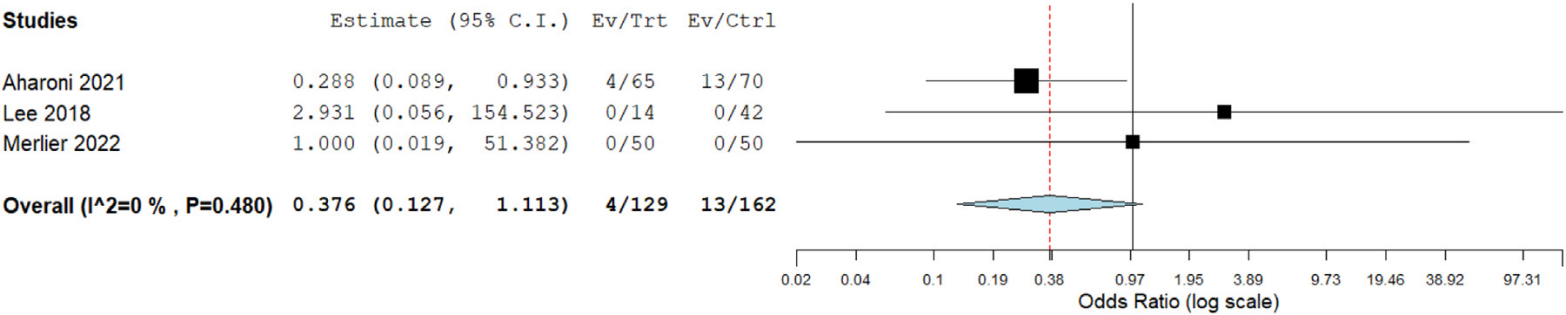
Meta-analysis of the rate of intraoperative complications.

Three studies reported intraoperative complications.^13^^,^^15^^,^^16^ We found no significant variation between both cohorts (OR=0.376 [0.127, 1.113]; *P*=.077). Data was homogeneous (*P*=.480); I²=0% (Figure 6).

#### Postoperative complications

**Figure 7:**
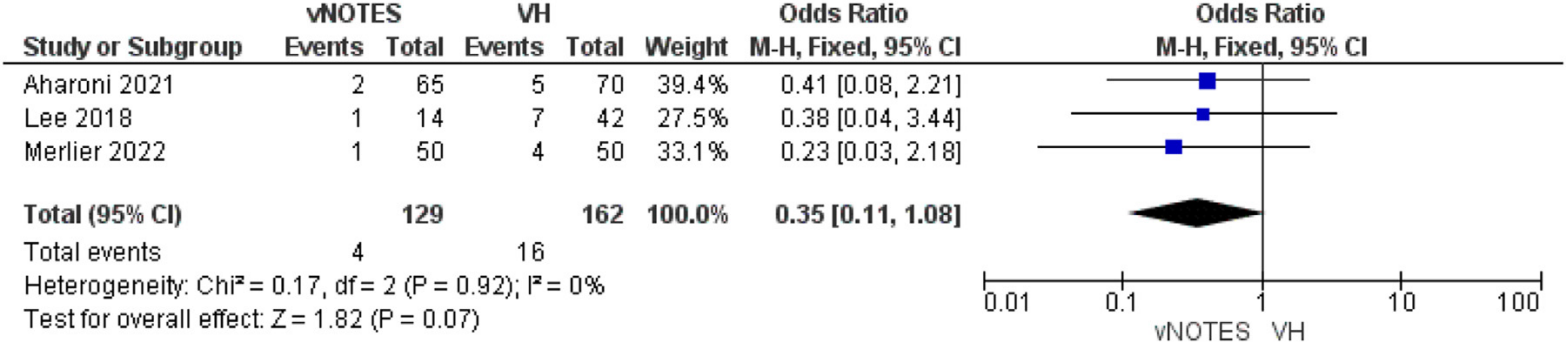
Meta-analysis of the rate of postoperative complications.

This outcome was investigated by 3 studies.^13^^,^^15^^,^^16^ The incidence of postoperative complication was similar in both techniques (OR=0.35 [0.11, 1.08]; *P*=.07). Pooled analysis was again homogeneous (*P*=.92); I²=0% (Figure 7).

## Comment

This study is the first systematic review and meta-analysis that compares hysterectomy by vNOTES with vaginal hysterectomy. There are many factors that surgeons depend on to choose the procedure of choice for hysterectomy, such as the size of the uterus, the indication of surgery, the surgeon's experience, and the co-morbidities of each patient. Each surgical procedure has a specific incision type, recovery time, perioperative consequences, and expected recovery process.^17^ vNOTES, in particular, is the newest of the MIS techniques, and has the potential to minimize tissue trauma and optimize perioperative outcomes.^17^^,^^18^ However, we have noted significant heterogeneity among the currently available studies evaluating this procedure's efficacy and safety. This heterogeneity may be attributed to the lack of surgical standardization due to the novelty and recent adaptation of this procedure by gynecologic surgeons.^19^

### Principal findings

Initially, our analysis showed no statistically significant difference between hysterectomy by vNOTES and conventional hysterectomy in all studied outcomes, including the duration of the operation, estimated blood loss, the duration of hospital stay, the VAS score after one day, intraoperative, and postoperative complications. However, we faced heterogeneity in some outcomes.

### Comparison with existing literature

In 2022, Merlier et al^16^ conducted a retrospective cohort study that aimed to evaluate vNOTES hysterectomy versus the conventional vaginal hysterectomy. They included 50 patients requiring hysterectomy for benign diseases in each cohort. Their analysis demonstrated no significant variation between both procedures concerning the surgical outcomes (*P*=.23), which is consistent with our findings. They also reported a similar success rate of outpatient management in both approaches (*P*=.85) and slightly higher rates of adnexectomy and salpingectomy in the vNOTES cohort. Salpingectomy and adnexectomy are essential additional procedures that are performed at the same time as hysterectomy to reduce the incidence of future ovarian malignancy.^20^ Although this additional procedure is important, it is considered challenging during the conventional vaginal hysterectomy. vNOTES may provide better visualization and easier access to the adnexa than conventional vaginal hysterectomy.^21^^,^^22^

Another retrospective study by Aharoni et al^13^ investigated the surgical and short-term perioperative outcomes of vNOTES hysterectomy versus conventional vaginal hysterectomy associated with uterosacral ligament suspension They concluded that vNOTES hysterectomy with uterosacral ligament suspension was associated with statistically significant shorter operation time, decreased intra-operative complications, and more duration of hospitalization compared with conventional vaginal hysterectomy. They also found comparable postoperative complications in both procedures. They theorized that the significant decrease in intraoperative ureteral obstruction in the vNOTES technique was because the ureters could be seen more clearly before suture placement through the uterosacral ligament.^23^

In 2023, Chaccour et al^24^ conducted a systematic review and meta-analysis to compare hysterectomy by vNOTES with laparoscopic hysterectomy in terms of efficacy, surgical outcomes, complications, and cost. Their analysis favored the vNOTES technique regarding operation time, recovery time, postoperative pain, and complications. They also reported no considerable variations in the frequency of perioperative problems, perioperative blood loss, postoperative change in hemoglobin levels, or need for transfusion. According to other studies, compared to total laparoscopic hysterectomy and conventional vaginal hysterectomy, the vNOTES procedure is associated with decreased need for blood transfusion, lower abdominal wall complications, better cosmesis, and improved postoperative pain and recovery.^2^^,^^8^^,^^17^ Additionally, vNOTES may provide more comfortable ergonomics for the surgeon and the assistants, allowing better surgeon satisfaction.^25^

### Strengths

Our study is the first we are aware of to statistically compare vaginal and vNOTES hysterectomy in a meta-analysis. Also, the risk of bias in the included studies was overall found to be low.

### Limitations

The main limitation of this study is the search did not retrieve RCTs with a low risk of bias. Also, because we used retrospective studies we cannot rule out the possibility of measurement bias. Unfortunately, we could not retrieve any relevant randomized controlled trials on this topic at this time and as a result we cannot exclude the possibility of publication bias. Several other limitations exist, including the different indications for hysterectomy among the included studies, which may affect the surgical and postoperative outcomes. Next, another potential limitation of this study is the small sample size and the heterogeneity observed in some outcomes. This heterogeneity may be attributed to the different indications of hysterectomy in the included studies. Lastly, our inclusion criteria included studies that compared other procedures in addition to hysterectomy (such as prolapse repair), as long as they were performed in both the vaginal in vNOTES groups. The efficacy of the vNOTES or vaginal techniques in the performance of these procedures could influence our overall data.

## Conclusion

Hysterectomy by vNOTES seems to be a reliable, safe, and effective alternative to conventional vaginal hysterectomy with no significant difference in surgical and postoperative outcomes. However, vNOTES may also provide more advantages, such as better visualization of the adnexa, better cosmesis, and less tissue trauma than the traditional vaginal hysterectomy. Additional high-evidence randomized clinical trials are required to establish the effective role of the vNOTES technique and determine superiority of either procedure in the treatment of women requiring hysterectomy for benign conditions.

## CRediT authorship contribution statement

**Greg J. Marchand:** Project administration, Formal analysis, Conceptualization. **Hollie Ulibarri:** Validation, Data curation. **Amanda Arroyo:** Formal analysis, Data curation. **Madison Blanco:** Formal analysis, Data curation. **Daniela Gonzalez Herrera:** Visualization, Validation, Investigation, Formal analysis, Data curation. **Brooke Hamilton:** Visualization, Validation, Supervision, Methodology, Formal analysis, Data curation. **Kate Ruffley:** Visualization, Validation, Supervision, Investigation, Formal analysis, Data curation. **Ali Azadi:** Writing – review & editing, Writing – original draft, Supervision, Conceptualization.
